# NRF2-directed PRPS1 upregulation to promote the progression and metastasis of melanoma

**DOI:** 10.3389/fimmu.2022.989263

**Published:** 2022-09-20

**Authors:** Guohang Xiong, Yu Feng, Xiaojia Yi, Xuedan Zhang, Xiaoyu Li, Lijuan Yang, Zihan Yi, Buqing Sai, Zhe Yang, Qiao Zhang, Yingmin Kuang, Yuechun Zhu

**Affiliations:** ^1^ Department of Biochemistry and Molecular Biology, School of Basic Medical Sciences, Kunming Medical University, Kunming, China; ^2^ Department of Pathology, The Second Affiliated Hospital of Kunming Medical University, Kunming, China; ^3^ Department of Medical Oncology, The Third Affiliated Hospital of Kunming Medical University (Tumor Hospital of Yunnan Province), Kunming, China; ^4^ Department of Pathology, The First Affiliated Hospital of Kunming Medical University, Kunming, China; ^5^ Department of Organ Transplantation, The First Affiliated Hospital of Kunming Medical University, Kunming, China

**Keywords:** PRPS1, NRF2, melanoma, proliferation, metastasis

## Abstract

Phosphoribosyl pyrophosphate synthetase 1 (PRPS1) is the first enzyme in the *de novo* purine nucleotide synthesis pathway and is essential for cell development. However, the effect of PRPS1 on melanoma proliferation and metastasis remains unclear. This study aimed to investigate the regulatory mechanism of PRPS1 in the malignant progression of melanoma. Here, we found PRPS1 was upregulated in melanoma and melanoma cells. In addition, our data indicated that PRPS1 could promote the proliferation and migration and invasion of melanoma both *in vitro* and *in vivo*. PRPS1 also could inhibit melanoma cell apoptosis. Furthermore, we found NRF2 is an upstream transcription factor of PRPS1 that drive malignant progression of melanoma.

## Introduction

The maximum proliferation ability of cells is limited by the abundance of their nucleotide library and the level and activity of different rate-limiting enzymes in the nucleotide synthesis pathway (1). Compared with normal cells, tumor cells exhibit a larger nucleotide pool, higher activity of the nucleotide anabolic pathway, and lower activity of the nucleotide catabolic pathway (1). PRPS1 belongs to the phosphoribosyl pyrophosphate synthetase (PRPS) family. PRPS consists of five members, namely, PRPS1, PRPS2, and PRPS3 (PRPS1L1) with catalytic activity, and PAP39 and PAP41 without catalytic activity (2). PRPS1 can catalyze ribose-5-phosphate (R5P) to 5-phosphoribosyl-1-pyrophosphate (PRPP), which is the first rate-limiting purine nucleotide (3, 4). Additionally, PRPP is a donor of R5P for the synthesis of pyrimidine. The activity of PRPS1 is regulated by ADP and AMP negative feedback (4–6) and chemical modification (3, 6).

Previously, it was reported that the PRPS1 gene mutation could lead to deafness (7, 8), female cerebellar ataxia (9), gout (10), diabetes insipidus, and white matter disease (11). Recently, it has been reported that aberrant expression or mutation of PRPS1 is closely related to a variety of cancers, such as accelerating the proliferation of colorectal cancer (12, 13), esophageal squamous cell carcinoma (14), neuroblastoma (15) and childhood neuroblastoma (16), glioblastoma multiforme (17), promoting tumor invasion and metastasis (12, 15), changing colorectal cancer (3, 13), brain tumor initiating cells (18) and purine metabolism, and enhancing the drug sensitivity of lymphoblastic leukemia (4, 19–21) and breast cancer (22). However, it is still elusive whether PRPS1 is related to the proliferation and metastatic progression of melanoma.

In addition, melanoma cells encounter considerable oxidative stress due to endogenous factors, such as mitochondrial respiration and melanogenesis, as well as exogenous factors, such as ultraviolet radiation and melanoma (23, 24). These oxidative stresses are largely regulated by nuclear factor (erythroid-derived-2)-like 2 (NRF2) (24). Therefore, previous studies have focused on the regulation of NRF2 on oxidative stress in melanoma. Recently, studies have shown that NRF2 is not only related to oxidative stress, but also to the nucleotide metabolism. For example, NRF2 can regulate nucleotide biosynthesis and redox homeostasis thereby promoting the recurrence of dormant breast cancer (25). NRF2 up-regulates the pentose phosphate pathway (PPP) enzyme, glucose-6-phosphate dehydrogenase (G6PD) and transketase (TKT) mediated nucleotide biosynthesis, thereby promoting the malignant progression of head and neck squamous cell carcinoma (HNSCC) (26). Also, NRF2 promotes nucleotide production in non-small cell lung cancer by regulating the expression of key serine/glycine biosynthetic enzymes (27).

However, the correlation between NRF2 and PRPS has not been reported, whether in tumors or other diseases. The role of NRF2 in the regulation of PRPS1 expression has not yet been revealed. In this study, we first confirmed that PRPS1 is highly expressed in melanoma. The abnormally high expression of PRPS1 promotes the growth and metastasis of melanoma *in vivo and in vitro*. In addition, we found that NRF2 is a PRPS1 transcription factor that can bind to the PRPS1 promoter and upregulate the expression of PRPS1. Our findings provide a theoretical basis for PRPS1 as a potential therapeutic target for melanoma.

## Materials and methods

### Cell culture

Human melanoma cell lines (A875 and SK-MEL-110) were purchased from the Cell Bank of the Chinese Academy of Science. All cells were maintained in DMEM (Life Technologies, Carlsbad, CA, USA) supplemented with 10% fetal bovine serum at 37°C in 5% CO_2_.

### Cell transfection

A875 and SK-MEL-110 cells were transduced with PRPS1 overexpression (overexpression vector LV-PRPS1 or the corresponding control (CON335)) or PRPS1 knockdown (shRNA vector LV- PRPS1-RNAi) and the corresponding control (CON313) for 48 h. A875 and SK-MEL-110 cells were transduced with NRF2 overexpression (overexpression vector LV-PRPS1 or the corresponding control (CON335)) or NRF2 knockdown (shRNA vector LV- PRPS1-RNAi) and the corresponding control (CON313) for 48 h. The lentivirus expression vectors were purchased from Ji Kai Gene Chemical Technology Co., Ltd. (Shanghai, China). Then, the cells were selected with different concentrations of puromycin until the GFP-positive signal of the cells was not less than 95% observed under the fluorescence microscope. The transfection efficacy was determined by qPCR and western blotting.

### Quantitative real-time PCR

Gene expression was evaluated by quantitative real-time PCR (qPCR). qPCR was performed according to the manufacturer’s instructions and was synthesized by real-time PCR (American Applied Biosystems) using SYBR Green (Roche, Switzerland). The PCR primer pairs used to amplify the target gene are shown in **Table 1**.

**Table 1:** the sequence of the primers for qPCR.

Gene	primer
PRPS1	F: 5’- -3’: CGTTGTTGATGCGAGAAA R: 5’- -3’: ATGGTGCTTGTGGGAGAT
cyclin E1	F: 5’- -3’: ACTCAACGTGCAAGCCTCG R: 5’- -3’: GCTCAAGAAAGTGCTGATCCC
CDK2	F: 5’- -3’: CCAGGAGTTACTTCTATGCCTGA R: 5’- -3’: TTCATCCAGGGGAGGTACAAC
P16	F: 5’- -3’: GGGTTTTCGTGGTTCACATCC R: 5’- -3’: CTAGACGCTGGCTCCTCAGTA
Bax	F: 5’- -3’: AGACACTCGCTCAGCTTCTTG R: 5’- -3’ CTTTTGCTTCAGGGTTTCATC
Bcl_2_	F: 5’- -3’: GTGCCTGCTTTTAGGAGACCGA R: 5’- -3’: GAGACCACACTGCCCTGTTGATC
Caspase-3	F: 5’- -3’: CATGGAAGCGAATCAATGGACT R: 5’- -3’: CTGTACCAGACCGAGATGTCA
NRF2	F: 5’- -3’: GAAAATCCATCTTCCTTCACTTG R: 5’- -3’: GAGTTTGCTTGCCCATTGTAA
U6	F: 5’- -3’: CTCGCTTCGGCAGCACA −3′ R: 5’- -3’: AACGCTTCACGAATTTGCGT

### Western blot

The cells were prepared in RIPA buffer (Solarbio, #R0020). A BCA™ Protein Assay kit (Applygen, #P1511) was used to determine the protein concentration. The proteins (40 μg/sample) were separated by different polyacrylamide gel electrophoresis, transferred to PVDF membranes (Millipore, #IPVH00010), and incubated with the corresponding primary antibody at 4°C overnight. Then, the membranes were incubated with the corresponding secondary antibodies at room temperature for 1 h and measured with a chemiluminescence reagent ECL kit (Advansia, #K-12045-D50).

The primary antibodies used in the experiment used were: anti-PRPS1 (Proteintech, #15549-1-AP), anti-cyclin E1 (Proteintech, 11554-1-AP), anti-CDK2 (Proteintech, 10122-1-AP), anti-P16 (Proteintech, #10883-1-AP), anti-Bax (Proteintech, 50599-2-Ig), anti-Bcl2 (Proteintech, 12789-1-AP), anti-Cleaved-caspeas3 (CST, #9664), anti-MMP2 (Abcam, ab37150), anti-MMP9 (Abcam, ab76003), anti-MMP13 (Proteintech, 18165-1-AP), anti-E-Cadherin (Proteintech, 20874-1-AP), anti-N-Cadherin (Proteintech, 22018-1-AP), anti-Vimentin (Proteintech, 10366-1-AP), anti-NRF2 (Abcam, ab89443), anti-β-actin (Bioss, bs-0061R), and Tubulin (Abcam, #ab7291). The secondary antibodies used in the experiment were anti-rabbit IgG (Abcam, #ab6721) and anti-mouse IgG (Jackson ImmunoResearch Laboratories, 115-035-003).

### Immunohistochemistry

The immunohistochemical assay was performed as previously described (28) using anti-PRPS1 (Proteintech, #15549-1-AP), anti-NRF2 (Abcam, ab89443) and anti-(Proteintech, #15549-1-AP) antibodies. Tissue microarrays (MME1004i) were purchased from xi,an Taibosi Biological Technology Co., Ltd. (Xian, China). Immunohistological assessment was performed as previously described (29).

### Hematoxylin and eosin staining

The lung tissue from metastatic mice was fixed in 4% paraformaldehyde for 24 h, dehydrated in different concentrations of graded ethanol, embedded and cut into 4 μm thick slices. The slices were baked at 55°C for 5 h and stained with hematoxylin (Solarbio, #G1140) and eosin (Solarbio, #G110).

### CCK8 assay

A875 and SK-MEL-110 cells with PRPS1 overexpression or knockdown and the corresponding control were inoculated in 96-well plates (800 cells/well) and cultured for 0 h, 12 h, 24 h, 36 h, 48 h and 72 h. The cells were treated with 10 μl CCK-8 (APEBIO, #k1018) at 37°C for 1 hour. The absorbance was assessed by a microplate reader (Thermo Scientific, #51119200) at 450 nm. The proliferation rate (fold) = the cell absorbance at each time points minus blank hole absorbance/cell absorbance at initial time.

### Colony formation assay

Stable melanoma cells were seeded into 6-well plates at a density of 500 cells/well and continuously cultured for two weeks. The cells were washed three times with PBS every three minutes. Then, the cells were fixed in 4% paraformaldehyde for 20 min, washed three times with PBS again, and stained using 3% crystal violet.

### EdU assay

The cells were stained with a BeyoClick EdU Cell Proliferation kit (Beyotime, #C0075S). A fluorescence microscope (Leica, #DM4B, × 200) was used to obtain high-quality images.

### Flow cytometry

Cell proliferation was assessed using flow cytometry. The cells were starved in serum-free DMEM for 24 h and then cultured in 10% FBS DMEM for 48 h. The cells were fixed in 75% ethanol for 24 h at 4°C, washed with PBS, treated with PI (Biotech, #FXP0211) for 15 min and detected by a PARTEC CyFlow Space flow cytometer.

Cell apoptosis was evaluated using flow cytometry. The cells were incubated with TNF-α+SM-164 (Beyotime, #C0006S) for 6 h. Cell apoptosis was detected using an apoptosis detection kit (Dojindo, #AD11) and a PARTEC CyFlow Space flow cytometer.

### TUNEL apoptosis assay

The cells were incubated with TNF-α+SM-164 (Beyotime, #C0006S) for 6 h. A TUNEL apoptosis assay kit (Beyotime, #C1090) was used to measure cell apoptosis. Images were obtained by using a fluorescence microscope (Leica, #DM4B).

### Wound healing assay

The cells were inoculated into 75 cm^2^ petri dishes and cultured with DMEM without serum overnight. Cells were wounded with a 20 μl pipette tip. The pictures were acquired at 0 h and 36 h after wounding using a fluorescence microscope (Leica, #DM4B). Wound closure (fold) = (the initial scratch area- the unhealed area after 36 hours of scratch)/the initial scratch area.

### Transwell migration assay and Transwell invasion assay

The cells were resuspended in serum-free medium and placed in the upper chamber of a Transwell filter (Corning, #3524). For the Transwell invasion assay, the upper Transwell was coated with 1:8 diluted matrix adhesive (BD, #356234) in advance. DMEM containing 15% FBS was added to the lower chambers. After 24 h, the cells were fixed with 4% paraformaldehyde for 15 min, stained with 3% crystal violet, washed with PBS, and photographed with a fluorescence microscope (Leica, #DM4B).

### Melanoma cell line xenograft model

The xenograft models were generated in 4- to 6-week-old female or male BALB/c nude mice (Department of Experimental Animals, Kunming Medical University). Animals (n=6/group) were injected with 150 µl of PBS containing 1×10^7^ cells subcutaneously into one side of the back and tail of the mice (the back: the corresponding control group, the tail: the PRPS1-overexpression group or PRPS1-knockdown group). After 42 days, the mice were sacrificed, and the tumors were collected and weighed. According to the experimental needs, the tumor was divided into three parts, which were used for western blotting, qPCR, and immunohistochemistry. All animal experiments were approved by the Institutional Animal Care and Use Committee of Kunming Medical University.

### Melanoma cell line metastatic model

Twenty-four female or male BALB/c nude mice (Department of Experimental Animals, Kunming Medical University) were randomly divided into four groups. Six mice in one group were injected with A875 cells with PRPS1 overexpression, A875 cells with PRPS1 knockdown and control cells. The mice were injected with 500 µl of PBS containing 2×10^7^ cells *via* the caudal vein. Forty-two days later, the mice were sacrificed, and the lung tissues were collected and photographed. According to the experimental needs, the lung tissues were divided into three parts, which were used for western blotting and qPCR, H&E staining, and immunohistochemistry. All animal experiments were approved by the Institutional Animal Care and Use Committee of Kunming Medical University.

### Luciferase assays

Plasmid transfection and luciferase activity were detected using a luciferase assay kit (Vigorous, #T002) according to the manufacturer’s protocol. *PRPS1-luc* and GL3-Basic-PRPS1 were purchased from QingKe Bio Technology (Wuhan, China). The Luciferase activity (fold) = (Firefly luciferase/Renilla luciferase ratio was calculated for each experimental group)/(Firefly luciferase/Renilla luciferase ratio was calculated for each the control group).

### Chromatin immunoprecipitation

The ChIP assay was carried out based on a previous report (30). ChIP assays were performed using a ChIP assay kit (Abcam, #ab500) according to the manufacturer’s instructions with the indicated antibody: anti-NRF2 (Abcam, ab89443).

### Statistical analysis

The data analysis was performed using GraphPad Prism 8 software. All results are expressed as the mean ± SD or mean ± standard error. P value <0.05 was regarded as statistically significant. One-way analysis of variance (ANOVA) and unpaired or paired-sample Student’s t test and mixed ANOVA were used to determine statistical significance.

## Results

### PRPS1 is highly expressed in melanoma and is linked to the malignant degree of melanoma

**Figure 1:**
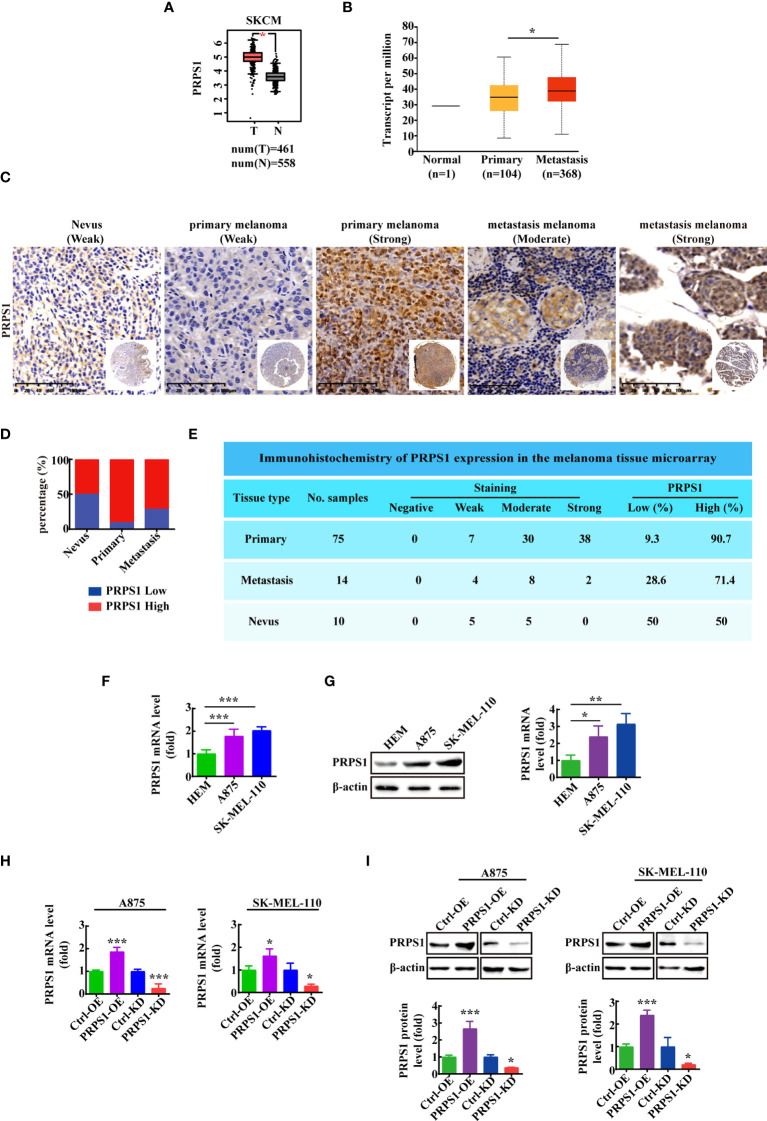
PRPS1 is highly expressed in melanoma and is related to the degree of malignancy of melanoma. **(A)** The expression of PRPS1 in normal tissue and melanoma tissue based on GEPIA database. **(B)** The expression of PRPS1 in normal tissue and primary melanoma and metastasis melanoma based on Ualcan database. **(C)** Representative images of PRPS1 expression in the melanoma tissue microarray are shown (200×). Scale bars=100μm. **(D)** Percentage of primary melanoma and metastasis melanoma with PRPS1-Low and PRPS1-High expression. **(E)** Immunohistochemistry of PRPS1 expression in the melanoma tissue microarray. **(F, G)** The mRNA and protein expression of PRPS1 in HEM cell and melanoma cells were analyzed by qPCR **(F)** and western blotting analysis **(G)**. **(H, I)** The stable PRPS1 overexpression and knockdown in A875 and SK-MEL-110 cells were established. The expression of PRPS1 were measured by Q-PCR **(H)** and western blotting **(I)** analysis. The data represent three independent experiments. The data as indicated the mean ± SD and was analyzed by student’s t-test. *p < 0.05; **p < 0.01; ***p < 0.001.

To investigate the role of PRPS1 in the proliferation and malignant progression of melanoma, we analyzed PRPS1 expression in melanoma based on the GEPIA database. In addition, we analyzed the correlation of PRPS1 with melanoma *in situ* and melanoma metastasis based on the UALCAN database. We found that the expression of PRPS1 was dramatically upregulated in melanoma (**Figure 1A**). It is worth noting that the expression of PRPS1 in metastatic melanoma patients was higher than that in primary melanoma patients (**Figure 1B**). Furthermore, an immunohistochemical (IHC) method was used to detect the expression of PRPS1 in melanoma tissues (melanoma *in situ* and metastatic melanoma) and normal nevi. The tissue specimens included 10 nevi, 76 primary melanomas and, 14 metastasis melanomas (one case of primary melanoma was not available). Representative images of the IHC staining analysis are shown in **Figure 1C**. Importantly, PRPS1 was highly expressed in 90.7% (68/75) of primary melanomas, 71.4% (10/14) of metastatic melanomas, and 50% (5/10) of nevus tissue samples (**Figure 1D**). More staining scores for PRPS1 in the tissue samples are summarized in **Figure 1E**. These results showed that the expression of PRPS1 was markedly increased in primary melanomas and metastatic melanomas.

Next, we detected the expression of PRPS1 in HEM, A875 and SK-MEL-110 melanoma cell lines. The mRNA and protein expression levels of PRPS1 in melanoma cell lines were higher than those in HEM cell lines (**Figures 1F, G**). To explore the function of PRPS1 in melanoma cells, we successfully established stable PRPS1 overexpression and knockdown in A875 and SK-MEL-110 melanoma cell lines (**Figures 1H, I**). The level of PRPS1 in the stably transfected A875 and SK-MEL-110 melanoma cells was measured by qPCR (**Figure 1H**) and western blot analysis (**Figure 1I**).

### PRPS1 promotes the proliferation of melanoma cells *in vitro*

**Figure 2:**
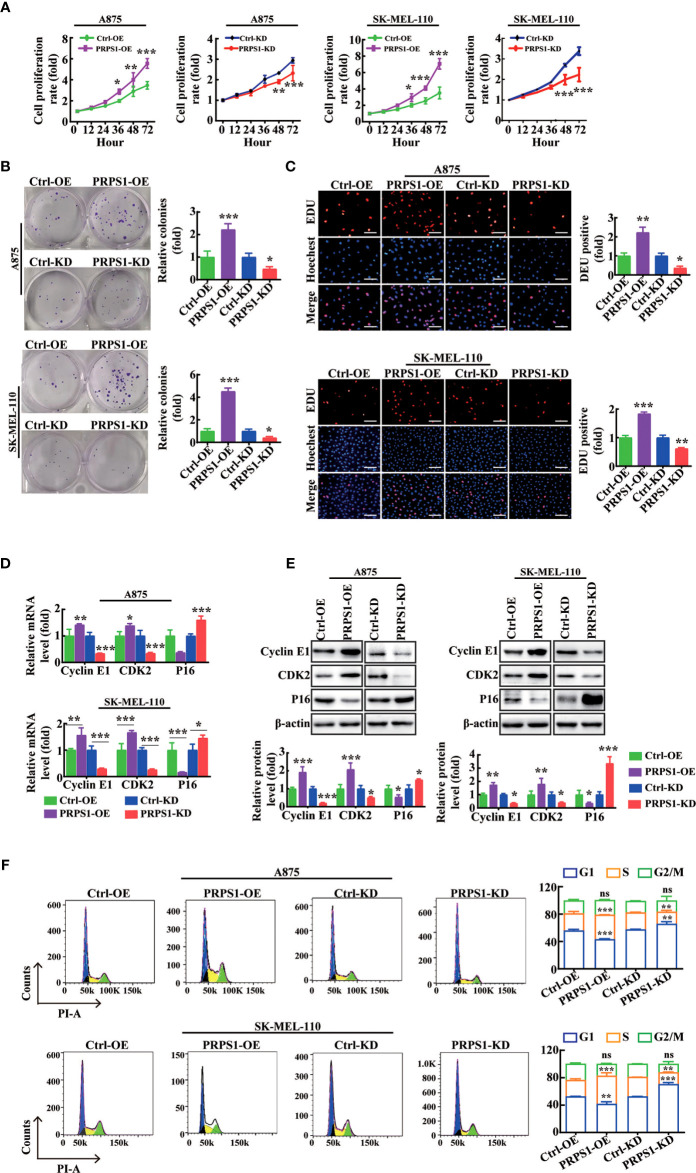
PRPS1 promotes melanoma cells proliferation *in vitro.* **(A–C)** The proliferation rate of A875 and SK-MEL-110 cells with PRPS1 overexpression or knock-down and the corresponding control cells were detected by MTS assay **(A)** and cells colony formation assay **(B)** and EDU method **(C)** scale bars = 50μm. **(D–F)** The effects of PRPS1 on the phase distribution of melanoma cell cycle at mRNA level, protein level and cell level were analyzed by qPCR **(D)**, western blotting **(E)** and flow cytometry **(F)**. The data represent three independent experiments. Each bar represents mean ± SD. p values were calculated using a student t-test (*p < 0.05, **p < 0.01, ***p < 0.001 vs. each control). ns mean no significant difference.

To confirm that PRPS1 drives the proliferation progression of melanoma, first MTS and cell colony formation assays and EdU staining were performed. The CCK8 results showed that stable overexpression of PRPS1 markedly promoted melanoma cell growth. In contrast, knockdown of PRPS1 reduced A875 and SK-MEL-110 melanoma cell proliferation (**Figure 2A**). In the plate colony formation assay, we found that the colony forming ability of melanoma cells overexpressing PRPS1 was significantly stronger than that of the control group, and the melanoma cells with PRPS1 knockdown showed the opposite results (**Figure 2B**). EdU staining further showed that the growth of melanoma cells overexpressing PRPS1 was significantly faster than that of the control group, but the proliferation of cells with PRPS1 knockdown was slower than that of the control group (**Figure 2C**). The results demonstrated that PRPS1 could promote the proliferation of melanoma cells *in vitro*.

Next, RT–PCR, western blotting, and flow cytometry assays were used to analyze the effects of PRPS1 on the cell cycle phase distributions of melanoma cells. The RT–PCR results showed that compared to the control, the mRNA levels of cell cycle proteins, such as cyclin E1 and CDK2, were enhanced in PRPS1-overexpressing melanoma cells, but the mRNA level of P16 was inhibited (**Figure 2D**). In PRPS1 knockdown cells (**Figure 2D**), the opposite was true. The western blotting results showed that the protein levels of cyclins such as cyclin E1 and CDK2 were increased in PRPS1-overexpressing melanoma cells, while the level of P16 was decreased compared with the control group, while the opposite was true in PRPS1 knockdown A875 and SK-MEL-110 cells (**Figure 2E**). Flow cytometry confirmed that overexpression of PRPS1 increased the number of S-phase and G_2_-phase cells and decreased the number of G_1_-phase cells (**Figure 2F**). Conversely, in PRPS1 knockdown cells, the proportion of S phase and G_2_ phase cells was decreased, and the proportion of G_1_ phase cells was increased (**Figure 2F**). These findings further suggest that PRPS1 is important for the proliferation of melanoma cells.

### PRPS1 inhibits apoptosis of melanoma cells

**Figure 3:**
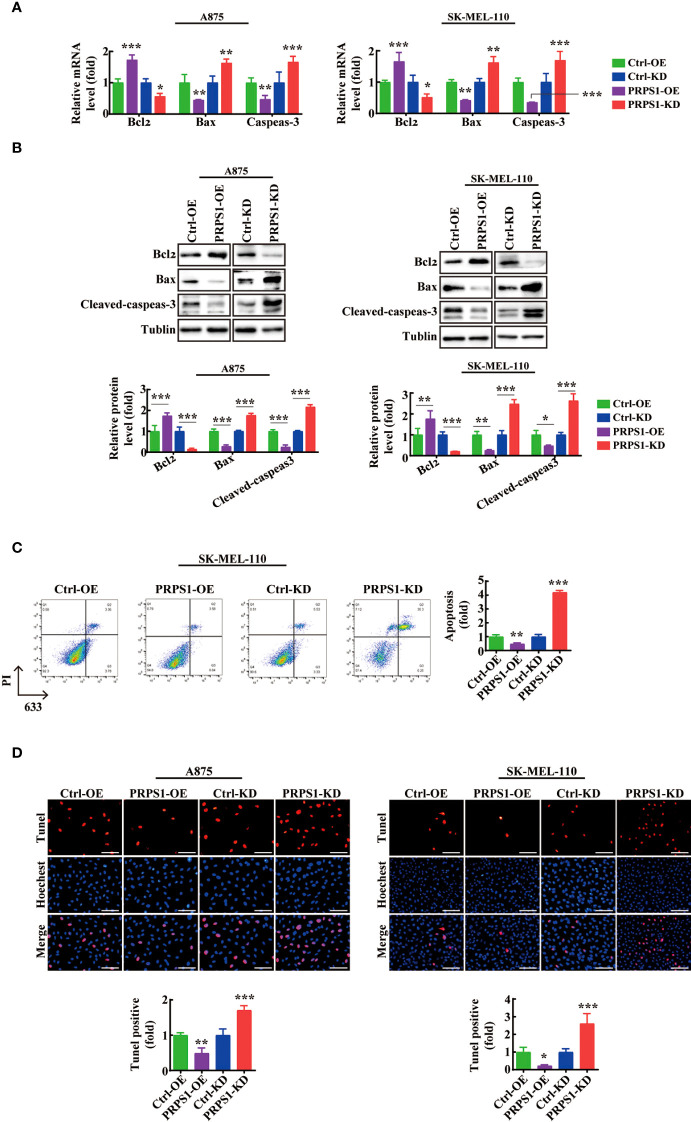
PRPS1 inhibits melanoma cell apoptosis. **(A, B)** The expressions of apoptosis related factors Bcl2 and Bax and Cleaved-caspeas-3 in A875 and SK-MEL-110 and the related control cells stably transfected with PRPS1 were detected by **(A)** qPCR and **(B)** western blotting analysis. **(C)** The anti-apoptotic ability of SK-MEL-110 cells overexpressing or knock-down PRPS1 and the control cells were evaluated by flow cytometry. **(D)** TUNEL analysis was used to analyze the anti-apoptotic ability of A875 (left) and SK-MEL-110 (right) over-expression or knock-down PRPS1 and the control cells. Scale bars=75μm. The data represent three independent experiments. Each bar represents mean ± SD. p values were calculated using a student t-test (*p < 0.05, **p < 0.01, ***p < 0.001 vs. each control).

To determine the relationship between PRPS1 expression and cell apoptosis in melanoma cells. We performed cell apoptosis detection. Based on qPCR and western blotting analysis, we found that the mRNA and protein levels of the apoptosis-related factor Bcl_2_ were significantly increased in A875 and SK-MEL-110 melanoma cells overexpressing PRPS1. Conversely, A875 and SK-MEL-110 melanoma cells with stable knockdown of PRPS1 exhibited lower mRNA and protein levels of the apoptosis-related factors Bax and cleaved caspase-3 than the controls (**Figures 3A, B**). In addition, by flow cytometry analysis, we found that the percentage of early apoptosis in melanoma cells with PRPS1 overexpression was lower than that in the control group. In contrast, the early apoptosis rate of PRPS1 knockdown melanoma cells was higher than that of the control group (**Figure 3C**). Furthermore, TUNEL staining showed that the number of TUNEL-positive cells in PRPS1-overexpressing melanoma cells was less than that in the control group. However, the number of TUNEL-positive cells in PRPS1-knockdown melanoma cells was greater than that in the controls (**Figure 3D**). These findings suggest that overexpression of PRPS1 reduces the apoptosis of melanoma cells and that knockdown of PRPS1 increases the apoptosis of melanoma cells.

### PRPS1 promotes the migration and invasion of melanoma cells

**Figure 4:**
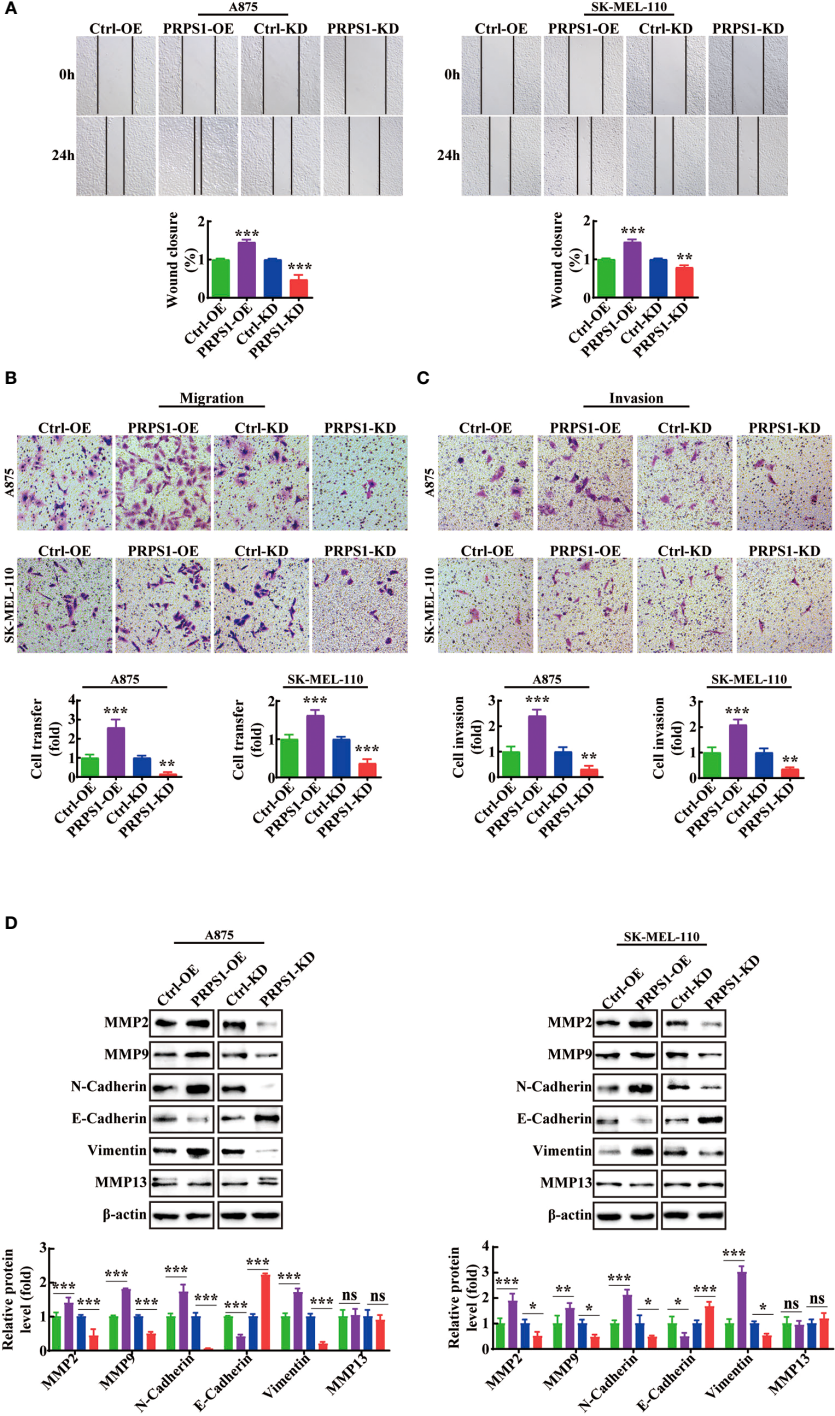
PRPS1 advances the migration and invasion of melanoma cells. **(A)** Representative scratch-wound images and the data analysis of PRPS1 over-expression and knock-down melanoma A875 and SK-MEL-110 cells 0h and 24h after scratch. **(B)** Representative images of transwell migration of stably transformed melanoma cells after 24h of starvation (top panel). Quantification of the number of migrating cells per field (bottom panel). **(C)** Representative images of transwell invasion assay pictured 24h (top panel). Quantification of the number of invasion cells per field (bottom panel). **(D)** The expression of EMT-associated proteins in the A875 and SK-MEL-110 melanoma cells with PRPS1 overexpression or knock-down and the control cells. The data represent three independent experiments. (*p<0.05, **p<0.01, *** *p*<0.001, ns mean no significant difference).

We thoroughly investigated the effect of PRPS1 on the invasion and malignant progression of melanoma. First, we conducted scratch and Transwell migration tests. We observed that the cell migration rate of PRPS1-overexpressing cells was much higher than that of the control group, but the cell migration rate of the PRPS1 knockdown group was lower than that of the control group (**Figures 4A, B**). Next, a Transwell invasion assay was performed to further estimate the invasion capability. In the Transwell invasion experiment, we also found that overexpression of PRPS1 promoted the invasion of melanoma cells, while knockdown of PRPS1 suppressed the invasion of melanoma cells (**Figure 4C**).

Moreover, western blotting was performed to assess the expression levels of EMT-associated proteins in the stable PRPS1 overexpression and knockdown A875 and SK-MEL-110 melanoma cell lines. As **Figure 4D** shows, PRPS1 promoted the expression of pro-invasion proteins, such as MMP2, MMP9, N-cadherin, and vimentin, and inhibited the expression of anti-invasion proteins, such as E-cadherin, in melanoma cells. Notably, knockdown or overexpression of PRPS1 did not cause changes in the protein level of MMP13 (**Figure 4D**). These results suggest that PRPS1 can markedly promote melanoma cell invasion and migration.

### PRPS1 drives melanoma tumor proliferation *in vivo*

**Figure 5:**
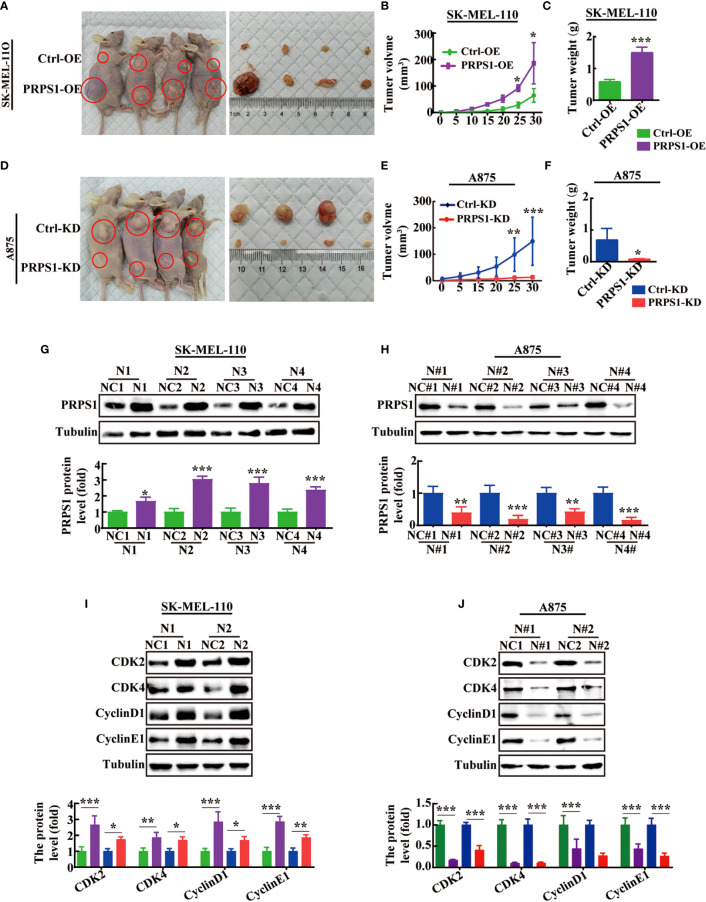
PRPS1 promotes melanoma cells proliferation *in vivo.* **(A–C)** BALB/c nude mice were injected with SK-MEL-110 cell that were stably transfected with PRPS1 overexpression and the control. Representative images of mice with control (top) and PRPS1-overexpressing (lower) xenograft tumors **(A)**. The tumor volume **(B)** and body weight **(C)** were measured. (n=6/group) **(D–F)** BALB/c nude mice were injected with A875 cell that were stably transfected with PRPS1 knockdown and the control. Representative photographs of mice with control (top) and PRPS1- knockdown (lower) xenograft tumors **(D)**. The tumor volume **(E)** and body weight **(F)** were measured. (n=6/group) **(G–J)** The protein level of PRPS1 **(G, H)** and the cell cycle related protein **(I, J)** levels of tumors in each xenograft tumors group were measured by western blot analysis. The data represent three independent experiments. The data related to tumor volume were statistically analyzed by two-way ANOVA, and the other data were analyzed by unpaired-sample Student’s *t* test. *P < 0.05, **P < 0.01, ***P < 0.001.

Next, we performed animal experiments to confirm whether the abnormal expression of PRPS1 affects the progression of melanoma proliferation *in vivo*. We found that the implantation of PRPS1 stably overexpressing A875 cells and PRPS1 knockdown SK-MEL-110 cells and control cells in BALB/c nude mice led to the occurrence of tumors *in vivo* (**Figures 5A**, **5D**). More importantly, PRPS1-overexpressing A875 cells significantly promoted tumor growth (**Figure 5A**). The tumors formed by PRPS1-overexpressing A875 cells were significantly earlier and faster, and larger than those in the control group (**Figures 5B, C**).

In contrast, our animal experiments demonstrated that PRPS1 cell knockdown was significantly detrimental to tumor growth (**Figures 5D–F**). Compared with control cells, the tumors induced by injection of PRPS1 knockdown cells grew slower and weighed less (**Figures 5E, F**).

In addition, we further compared the expression of PRPS1 in subcutaneous tumor tissues of nude mice by western blotting. We found that the protein expression of PRPS1 in the tumors was positively correlated with the tumor volume (**Figures 5G, H**). We also measured the protein levels of cell cycle-related proteins in the tumors, as **Figures 5I, J** show that CDK2, CDK4, cyclin D1, and cyclin E1 levels were significantly upregulated or reduced in PRPS1-overexpressing or PRPS1-knockdown tumors compared to the corresponding controls. These results suggest that PRPS1 promotes melanoma growth *in vivo*.

### PRPS1 promotes malignant melanoma tumors *in vivo*

**Figure 6:**
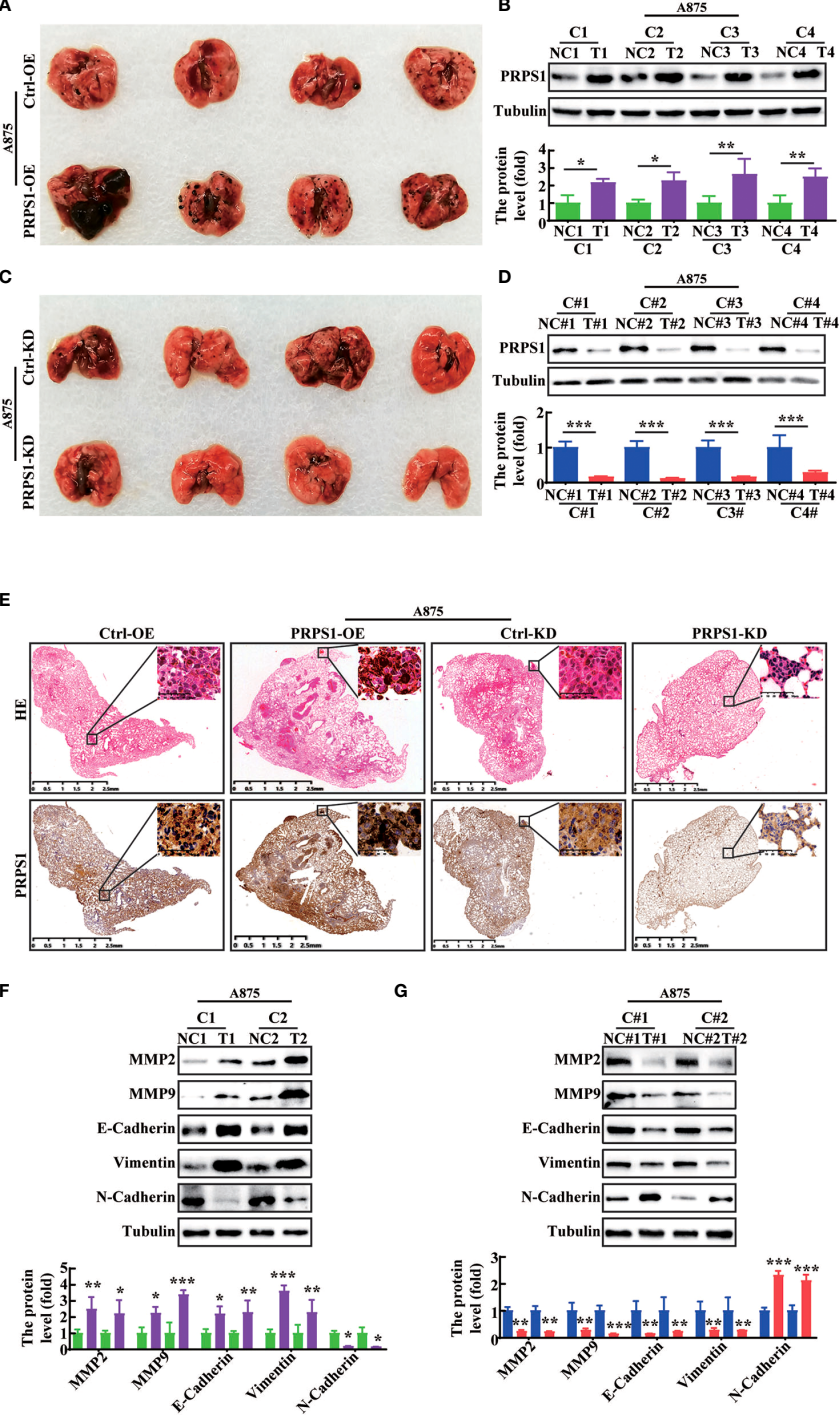
PRPS1 promotes melanoma cells metastasis *in vivo.* **(A–D)** *In vivo* experimental lung metastasis assay of A875 cells stably overexpressing **(A)** or knocking down PRPS1 **(C)**. The cells were injected into BALB/c nude mice *via* tail vein. Representative images of mice with the corresponding control (top) and PRPS1-overexpression/PRPS1-kockdown (lower) lungs metastasis. The expression of PRPS1 in PRPS1-overexpression and the control metastatic tumors **(B)**, as well as in the PRPS1-kockdown and the corresponding control metastatic tumors were compared by western blot analysis **(D)**. (n=6/group) **(E)** Morphological feature of the lung metastasis by HE staining and IHC in each metastatic tumor group. **(F, G)** The migration and invasion related protein levels in each metastatic tumor group were measured by western blot analysis. The data represent three independent experiments. Statistical analysis was carried out with unpaired-sample Student’s *t* test. *P < 0.05, **P < 0.01, ***P < 0.001.

To further confirm whether PRPS1 could promote tumor malignancy *in vivo*, we injected PRPS1-overexpressing or PRPS1-knockdown A875 cells and the corresponding control cells *via* the caudal vein to establish a metastatic tumor model in BALB/c nude mice. The incidence of lung metastasis in BALB/c nude mice injected with PRPS1-overexpressing A875 cells was significantly higher than that in the control group, but the incidence of lung metastasis in BALB/c nude mice injected with PRPS1-knockdown A875 cells was significantly lower than that in the control (**Figures 6A**, **6C**). We detected the protein expression of PRPS1 in lung metastasis tumors. Notably, western blotting demonstrated that the higher the expression of PRPS1 was, the stronger the ability of melanoma cells to metastasize (**Figures 6B**, **6D**). HE staining and IHC staining of lung tissue sections showed that BALB/c nude mice carrying A875 melanoma cells with PRPS1 overexpression had significantly increased formation of lung-specific metastases, and the expression level of PRPS1 was positively correlated with the number of tumor foci compared with BALB/c nude mice bearing control cells (**Figure 6E**). The opposite was true in BALB/c nude mice carrying PRPS1 knockdown A875 cells (**Figure 6E**). Meanwhile, we also detected the protein expression levels of cell migration- and invasion-related factors in nude mice, including MMP2, MMP9, E-cadherin, N-cadherin, and vimentin. The results demonstrated that overexpression of PRPS1 promoted the expression of EMT-related proteins, but knockdown of PRPS1 inhibited the expression of EMT-related proteins (**Figures 6F, G**). The above results indicate that PRPS1 promotes malignant melanoma tumors *in vivo.*

Taken together, the results suggest that PRPS1 drastically promotes the potential for tumor proliferation, malignancy, and metastasis of melanoma *in vitro* and *in vivo*.

### PRPS1 is upregulated by NRF2 and acts as a prominent determinant of melanoma proliferation and malignancy progression

We further analyzed the mechanism by which PRPS1 regulates the malignant progression of melanoma. Nuclear factor (erythroid-derived-2)-like 2 (NRF2) is a transcription factor that is known to play a pivotal role in the pentose phosphate pathway (PPP) of glioblastoma (31), breast cancer cells (32), head and neck cancer (26), human hepatoma cells (33), and colon cancer (34) and to affect cell metabolic reprogramming. PRPS1 acts as an enzyme that catalyzes R5P to PRPP, thus participating in the PPP (3, 4). Therefore, we investigated whether NRF2 affects the malignant progression of melanoma by regulating PRPS1.

**Figure 7:**
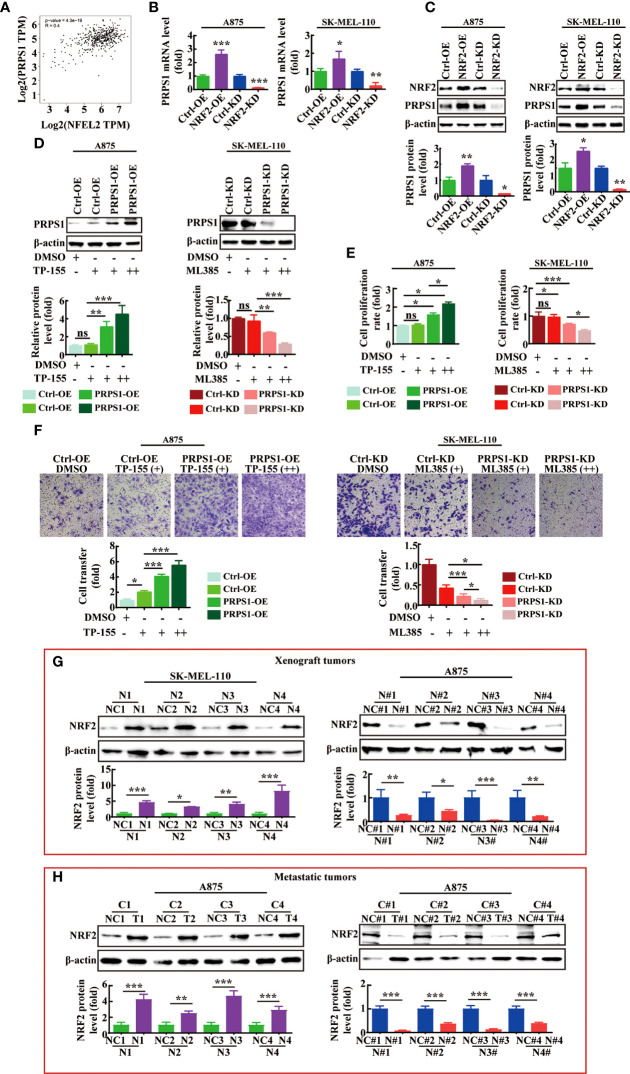
NRF2 is positively correlated with PRPS1 expression in melanoma. **(A)** Spearman correlation analysis of PRPS1 and NRF2 mRNA expression levels was performed in melanoma and normal skin tissues through GEPIA website. **(B, C)** The mRNA **(B)** and protein **(C)** level of PRPS1 were analyzed in the stably NRF2 overexpression or knockdown A875 and SK-MEL-110 and that the control cells. **(D–F)** The stably PRPS1 overexpression A875 and the control cells were treated with TP-155 (NRF2 activator) for 24h. The stably PRPS1 knockdown SK-MEL-110 and the control cells were incubated with ML385 (NRF2 inhibitor) for 24h. The expression of PRPS1 was detected by using western blot in each group cells **(D)**. The cell proliferation rate was detected by MTS assay **(E)**. The rate of migration was measured by transwell assay **(F)**. TP-155 (+): 0.05uM, TP-155 (++): 0.1uM. ML385 (+): 2.5uM, ML385 (++): 5uM. The cell migration ability was evaluated by transwell migration **(F)**. TP-155 (+): 10nM, TP-155 (++): 25nM. ML385 (+): 1uM, ML385 (++): 2uM. **(G, H)** The expression of NRF2 in the xenograft tumors **(G)** and the lung metastases of nude mice **(H)** were detected by western blot analysis. Statistical analysis was carried out with unpaired-sample Student’s *t* test or two-way ANOVA. *P < 0.05, **P < 0.01, ***P < 0.001, ns mean no significant difference.

First, through analysis of the GEPIA website, we found that the level of PRPS1 gene expression was positively correlated with NRF2 (PRPS1-NRF2: Pearson correlation=0.4, p=3.4e-19) (**Figure 7A**). Next, we detected the mRNA and protein levels of PRPS1 in A875 and SK-MEL-110 cells with NRF2 overexpression and PRPS1 knockdown. The results showed that in A875 and SK-MEL-110 cells, stable overexpression of NRF2 increased the mRNA and protein levels of PRPS1, but knockdown of NRF2 markedly reduced the mRNA and protein levels of PRPS1 (**Figures 7B, C**). Furthermore, PRPS1-overexpressing A875 cells and control cells were incubated with 0.05 uM or 0.1 uM bardoxolone methyl (NRF2 activator, TP-155), PRPS1-knockdown SK-MEL-110 cells and control cells were treated with 2.5 uM or 5 uM ML385 (NRF2 inhibitor), and the results reconfirmed that the protein expression of PRPS1 was decreased in the cells with NRF2 inhibition and *vice versa* (**Figure 7D**). More importantly, the increase or decrease of PRPS1 expression was positively correlated with the dose of NRF2 activator or inhibitor.

However, it was unclear whether NRF2 could affect the proliferation and metastasis of melanoma cells by regulating the expression level of PRPS1. We used MTS analysis to evaluate the effect of NRF2 on stable cell proliferation. As shown in **Figure 7E**, the NRF2 activator significantly increased the proliferation rate of PRPS1-overexpressing melanoma cells, while the NRF2 inhibitor significantly decreased the proliferation rate of PRPS1-knockdown melanoma cells. Next, we evaluated whether NRF2 could influence the effects of PRPS1 on melanoma cell metastasis. We observed that the cell migration ability was improved in stable PRPS1-overexpressing A875 cells after treatment with an NRF2 activator (**Figure 7F**, left). In contrast, the NRF2 inhibitor significantly suppressed the ability of PRPS1 knockdown PRPS1 SK-MEL-110 cell migration (**Figure 7F**, right). It is worth noting that the NRF2 activator/NRF2 inhibitor has a significant dose-response relationship in promoting/inhibiting the proliferation, invasion and migration of PRPS1 overexpression/PRPS1 knock-down melanoma cell.

We further compared the expression of NRF2 in the animal models. We found that the expression of NRF2 was positively correlated with the expression of PRPS1 in the subcutaneous tumors (**Figure 7G**) and the lung metastases of nude mice (**Figure 7H**). The levels of PRPS1 and NRF2 in the tumors were positively correlated with the melanoma tumor volumes and the degree of melanoma metastasis in each group.

In conclusion, these results demonstrate that PRPS1 promotes the proliferation, malignancy, and metastasis of melanoma, which may be related to NRF2.

### NRF2 bound to PRPS1 is crucial for PRPS1 transcription

**Figure 8:**
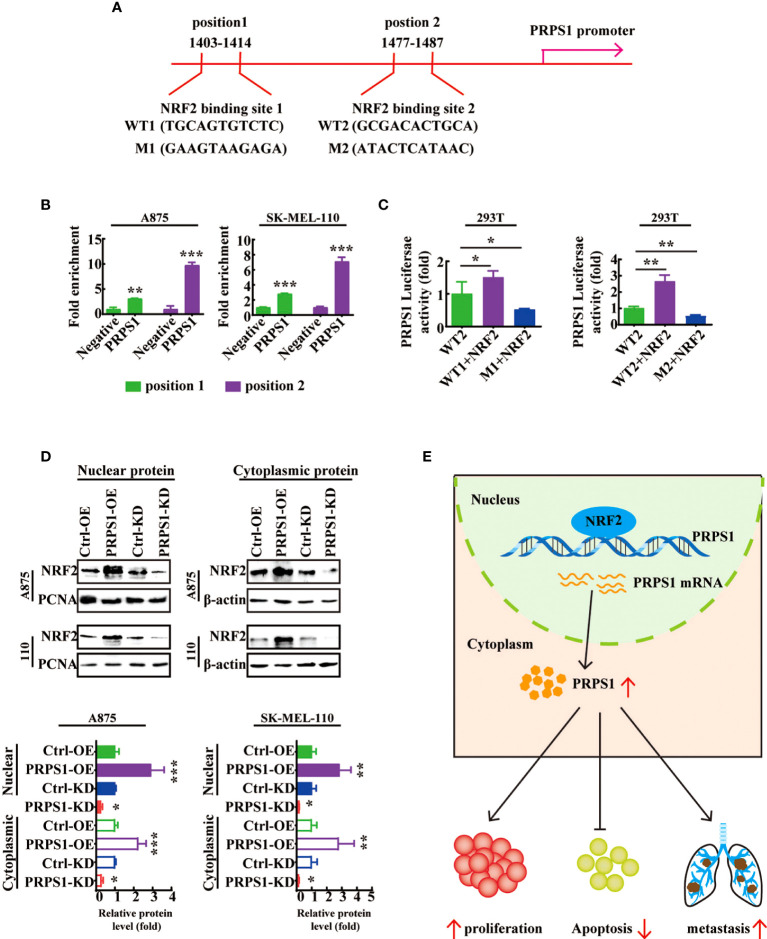
NRF2 is a transcription factor of PRPS1-mediated malignant progression of melanoma. **(A)** Consensus binding sites for NRF2 on the PRPS1 promoter was analyzed by JASPAR database. **(B)** ChIP assay was performed to assess NRF2 in the PRPS1 promoter region in A875 and SK-MEL-110 cells. **(C)** 293T cells were transfected with corresponding plasmids for 48h. Luciferase dual reporter assays were performed to measure the luciferase activity of PRPS1-luc. **(D)** NRF2 expression in nuclei and cytoplasm of A875 and SK-MEL-110 cells overexpressing or knockdown PRPS1. **(E)** Proposed model of the relationship between PRPS1 and NRF2 in melanoma cells. The data represent three independent experiments. The data represent three independent experiments. Each bar represents mean ± SD. *p* values were calculated using a student t-test (*p < 0.05, **p < 0.01, ***p < 0.001 vs. the control).

We further explored the mechanisms by which NRF2 regulates PRPS1 in melanoma cells. JASPAR database analysis showed that the transcriptional regulatory region of PRPS1 contains two NRF2 binding sites (**Figure 8A**). The ChIP–qPCR results showed that NRF2 was recruited to PRPS1 primer 1 (-1403-1414), which was 3.1 times that of the negative control, and PRPS1 primer 2 (1477-1487), which was 9.7 times that of the negative control in A875 melanoma cells (**Figure 8B**, left). In 110 melanoma cells, the abundance of NRF2 combined with PRPS1 primer 1 (1403-1414) was increased by 2.8 times and that combined with PRPS1 primer 2 (1477-1487) was increased by 7.1 times (**Figure 8B**, right). Furthermore, a luciferase assay showed that PRPS1-luc activity was increased in both 293T cells cotransfected with PRPS1 promoter1 wild-type/promoter2 wild-type and NRF2 overexpression plasmids (**Figure 8C**, left). In contrast, PRPS1-luc activity was decreased in both 293T cells cotransfected with PRPS1 position 1-mutated/position 2-mutated and NRF2 overexpression plasmids (**Figure 8C**, right). These results reveal that NRF2 is involved in directing PRPS1 expression in melanoma. We further investigated the relationship between PRPS1 and NRF2. We detected the protein levels of NRF2 in the nuclei and cytoplasm of A875 and SK-MEL-110 cells with overexpression or knockdown of PRPS1 and found that PRPS1 overexpression increased the NRF2 levels in the nuclei and cytoplasm and that knockdown of PRPS1 decreased the NRF2 levels in the nuclei and cytoplasm. (**Figure 8D**).

These results indicate that the transcription factor NRF2 can bind to the PRPS1 promoter and increase the transcription of PRPS1 to advance the proliferation, migration, and invasion of melanoma (**Figure 8E**).

## Discussion

Tumor cells, including melanoma, are highly dependent on *de novo* biosynthesis of purine and pyrimidine nucleotides (35, 36). The researchers found that the levels of xanthine, purine, pyrimidine, AMP, ADP, ATP, and UDP in the clonally expanded cells of metastatic lymph nodes in melanoma patients were significantly increased (37). We found that the mitochondrial oxidative phosphorylation pathway and purine biosynthesis were abnormally active in melanoma cells (unpublished data). The PPP pathway is often upregulated in cancer cell lines, enabling cancer cells to obtain a large amount of R5P for purine nucleotide and pyrimidine synthesis (1, 38, 39). We previously demonstrated that G6PD, the key enzyme of the PPP, is upregulated, and its enzyme activity is increased in melanoma, which can promote the proliferation of melanoma cells and inhibit apoptosis (40, 41).

PRPS1 catalyzes R5P to 5-phosphoribosyl-1-pyrophosphate, which is the first step of *de novo* nucleotide synthesis. Previous reports indicated that knockdown of PRPS1 strongly inhibited neuroblastoma cell proliferation (15). PRPS1 is upregulated by KHK-A and promotes the proliferation of esophageal squamous cells (14). CDK1 upregulates PRPS1 activity by phosphorylating PRPS1(183), so PRPS1 cell cycle-dependent phosphorylation promotes nucleotide synthesis in colon cancer (3). The lncRNA lymphocytic leukemia 1 (DLEU1), targeting miR-320b/PRPS1, promotes the proliferation, migration, and invasion and reduces the apoptosis of colorectal cancer (12). The PRPS1 mutation drove thiopurine resistance in childhood acute lymphoblastic leukemia (4, 19). In this study, our data demonstrated that PRPS1 is highly expressed in melanoma tissues and melanoma cell lines (**Figure 1C-G**). We also demonstrated that overexpression of PRPS1 promoted melanoma tumor proliferation, migration, and invasion *in vitro* and *in vivo* and inhibited melanoma cell apoptosis. In contrast, knockdown of PRPS1 suppressed proliferation, migration, and invasion while advancing apoptosis in melanoma (**Figures 2**, **6**). We first showed that PRPS1 could promote the growth, migration, and invasion of melanoma and prevent melanoma cell apoptosis.

A report pointed out that c-MYC is a transcription factor of PRPS1 in neuroblastoma (18). However, another study confirmed that in c-MYC-overexpressing malignant lymphoma cells, the gene expression of PRPS2 rather than PRPS1 is strongly regulated at the translational level to regulate purine synthesis (5). However, the mechanism by which PRPS1 regulates the malignant progression of melanoma remains unclear.

Our study found that NRF2 can regulate the transcription of PRPS1 and then regulate the proliferation and metastasis of melanoma. NRF2 is considered a marker of cancer and plays a role in tumor promotion and tumor suppression in different cancers (42). Research confirmed that knockdown of NRF2 led to reduced growth of melanoma cells (43). NRF2 promotes the migration and invasion of BRAF mutant melanoma cells (44). In our study, we confirmed that NRF2 could promote the transcription of PRPS1 (**Figure 7D**). The ChIP and luciferase assay data indicated that NRF2 binds to the PRPS1 promoter (**Figures 8B, C**). In addition, after SK-MEL-110 cells with stable PRPS1 overexpression were treated with an NRF2 activator and PRPS1 knockdown PRPS1 A875 cells were incubated with an NRF2 inhibitor, we reconfirmed that abnormal PRPS1 could promote the proliferation, migration and invasion of melanoma cells through NRF2-activated transcription (**Figures 7D, E**).

As a transcription factor, NRF2 is also the most potent effector of the oxidative stress response (43, 45). Because melanoma shows high oxidative stress in both the intracellular and tumor microenvironments, NRF2 is involved in this process. Therefore, previous studies have paid more attention to the regulation of NRF2 on oxidative stress in melanoma. Intermittent hypoxia promoted melanoma lung metastasis through oxidative stress in a mouse model of obstructive sleep apnea (46). Mitochondrial glycerol-3-phosphate dehydrogenase (mGPDH) inhibits melanoma migration, and invasion by suppressing NRF2 and downstream oxidative signals (47). Our study demonstrates for the first time that NRF2, as a transcription factor, can regulate the transcription of PRPS1, an important enzyme in nucleotide metabolism.

## Conclusion

Our study demonstrated that PRPS1 is highly expressed in melanoma and promotes melanoma proliferation and metastasis and decreases melanoma cell apoptosis. Moreover, abnormal expression of PRPS1 occurred *via* NRF2-mediated upregulation. This is the first study to provide data by systematically analyzing the function and regulatory mechanism of PRPS1 in melanoma. Targeting purine nucleotide metabolism may become a new strategy for melanoma therapy.

## Data availability statement

The datasets presented in this study can be found in online repositories. The names of the repository/repositories and accession number(s) can be found in the article/supplementary material.

## Ethics statement

All animal experiments were reviewed and approved by the Institutional Animal Care and Use Committee, Kunming Medical University.

## Author contribution

YZ and YK contributed to the design of experiments and finalization of the manuscript. GX performed experiments and wrote the manuscript. YF and XY and XZ and XL did the animal study. LY and ZiY conducted *in vitro* experiments. BS and ZhY and QZ participated in analyzed data. All authors contributed to the article and approved the submitted version.

## Funding

The research leading to these results has received funding from National Natural Science Foundation of China (No.31960200, No.31660246, No.82160540).

## Conflict of interest

The authors declare that the research was conducted in the absence of any commercial or financial relationships that could be construed as a potential conflict of interest.

## Publisher’s note

All claims expressed in this article are solely those of the authors and do not necessarily represent those of their affiliated organizations, or those of the publisher, the editors and the reviewers. Any product that may be evaluated in this article, or claim that may be made by its manufacturer, is not guaranteed or endorsed by the publisher.
